# The Combined Effects of Hospital and Surgeon Volume on Short-Term Survival after Hepatic Resection in a Population-Based Study

**DOI:** 10.1371/journal.pone.0086444

**Published:** 2014-01-22

**Authors:** Chun-Ming Chang, Wen-Yao Yin, Chang-Kao Wei, Cheng-Hung Lee, Ching-Chih Lee

**Affiliations:** 1 Department of Surgery, Buddhist Dalin Tzu Chi General Hospital, Chiayi, Taiwan; 2 Department of Otolaryngology, Buddhist Dalin Tzu Chi General Hospital, Chiayi, Taiwan; 3 Center for Clinical Epidemiology and Biostatistics, Buddhist Dalin Tzu Chi General Hospital, Chiayi, Taiwan; 4 School of Medicine, Tzu Chi University, Hualian, Taiwan; University Hospital Heidelberg, Germany

## Abstract

**Background:**

The influence of different hospital and surgeon volumes on short-term survival after hepatic resection is not clearly clarified. By taking the known prognostic factors into account, the purpose of this study is to assess the combined effects of hospital and surgeon volume on short-term survival after hepatic resection.

**Methods:**

13,159 patients who underwent hepatic resection between 2002 and 2006 were identified in the Taiwan National Health Insurance Research Database. Data were extracted from it and short-term survivals were confirmed through 2006. The Cox proportional hazards model was used to assess the relationship between survival and different hospital, surgeon volume and caseload combinations.

**Results:**

High-volume surgeons in high-volume hospitals had the highest short-term survivals, following by high-volume surgeons in low-volume hospitals, low-volume surgeons in high-volume hospitals and low-volume surgeons in low-volume hospitals. Based on Cox proportional hazard models, although high-volume hospitals and surgeons both showed significant lower risks of short-term mortality at hospital and surgeon level analysis, after combining hospital and surgeon volume into account, high-volume surgeons in high-volume hospitals had significantly better outcomes; the hazard ratio of other three caseload combinations ranging from 1.66 to 2.08 (p<0.001) in 3-month mortality, and 1.28 to 1.58 (p<0.01) in 1-year mortality.

**Conclusions:**

The combined effects of hospital and surgeon volume influenced the short-term survival after hepatic resection largely. After adjusting for the prognostic factors in the case mix, high-volume surgeons in high-volume hospitals had better short-term survivals. Centralization of hepatic resection to few surgeons and hospitals might improve patients’ prognosis.

## Introduction

The discussion about the association between volume and mortality after the high-risk surgical procedures is ongoing. High-volume hospitals have better long-term survival rates for some operations [1], [2]. Numerous studies have explored the association between surgeon volume and mortality for certain procedures [3],[4],[5],[6], but few addressed on the relationship between surgeon volume for hepatic resection and short-term mortality [7],[8],[9]. Besides, most volume-outcome relationship studies explored the association between the two at hospital or surgeon level.

Hepatic surgery is not an unusual operation and is now performed at many hospitals worldwide [10]. In Asia, hepatocellular carcinoma (HCC) has a high prevalence [11]. Due to donor shortage remains a main problem in Asia, hepatic resection being considered the first line curative methods for some patients with HCC [12],[13]. In addition, intra-hepatic cholangiocarcinoma, metastatic malignancies, and benign diseases, such as, trauma, intra-hepatic bile duct stone and benign tumors, also require hepatic resection [10].

However, a general limitation of most of the available studies, and those addressing survival after hepatic resection, is the lack of important prognostic factors, such as, indication for surgery, comorbidities, hepatitis/cirrhosis and cirrhosis-related complications and the combined effect of hospital and surgeon volume, which makes it difficult to take into account the relative effects of these correlated factors [7],[8],[9].

Therefore, by taking the known prognostic factors into consideration, the purpose of this study was to explore the combined effects of hospital and surgeon volume and the association between different surgeon volumes in high- and in low-volume hospitals in relation to short-term survival after hepatic resection by using a population-based national database of Taiwan patients between 2002 and 2006.

## Materials and Methods

### Ethics Statements

This study was initiated after being approved by the Institutional Review Board of the Buddhist Dalin Tzu Chi General Hospital, Taiwan. Because the identification numbers and personal information of the individuals included in the study were not included in the secondary files, the review board stated that written consent from patients was not required.

### Patients and Study Design

We used data between 2002 and 2006 from the National Health Insurance (NHI) Research Database, which covered medical benefit claims for over 23 million people in Taiwan (approximately 99 percent of Taiwan’s population) [14]. Taiwan’s NHI has the characteristics of universal insurance coverage, providing comprehensive services, and a single-payer system with the government as sole insurer. The database was monitored for completeness and accuracy by Taiwan’s Department of Health. Patients who underwent hepatic resection for cancer disease (HCC, cholangiocarcinoma, metastatic malignancy), and benign disease (eg, trauma, intra-hepatic bile duct stone, benign tumors) between 2002 and 2006 were included. A total of 13,159 patients were identified. The mortality was identified from the National Register of Deaths Database. Hospitals and surgeons were sorted using similar methods as previous studies [15],[16]. The method for defining high and low hospital and surgeon volume of hepatic resection was: (1) Hospitals were categorized by their total patient volume by using unique hospital identifiers in this database. The 13,159 patients were sorted into two approximately equal groups based on the cumulative hospital volume of hepatic resections performed [7],[8]. The cumulative hospital volume of 245 and more cases was defined as high-volume hospital. By this definition, there were 13 high-volume centers for hepatic resection. (Table S1 in Appendix S1). (2) Surgeons were categorized by their total patient volume by using unique identifiers in this database. We initially divided patients into two approximately equal groups by cumulative surgeon volume, but many surgeons in Taiwan performed few hepatic resections annually, the volume cutoff was low. Surgeon volume cutoff was therefore chosen as roughly one-third of all patients undergoing hepatic resection by high-volume surgeons [9]. The cumulative surgeon volume of 25 and more cases was defined as high-volume surgeon. By this definition, there were 59 high-volume surgeons. (Table S2 in Appendix S1).

### Measurements

The key dependent variable of interest was 30-day, 3-month and 1-year survival of these patients. Mortality was the outcome measure. Overall mortality included all causes of death occurring after the surgery. The key independent variables were the hospital volume, surgeon volume and the combination of surgeon and hospital volume, which were sorted into four groups based on volume (high-volume surgeons in high-volume hospitals, low-volume surgeons in high-volume hospitals, high-volume surgeons in low-volume hospitals, and low-volume surgeons in low-volume hospitals). Patient demographics included age, gender, indication for surgery, comorbidity, surgical procedure (major: lobectomy or more; minor:<lobectomy), and individual socioeconomic status (SES), geographic region, and urbanization level of residence. The comorbidities included hypertension, ischemic heart disease, arrhythmia, cerebrovascular disease, chronic obstructive pulmonary disease and associated conditions including chronic bronchitis and asthma, renal disease, and diabetes. Patients’ severity of underlying liver disease was evaluated by presence or absence of hepatitis/cirrhosis and cirrhosis-related complications. The cirrhosis-related complications included portal hypertension, esophageal/gastric varices bleeding, ascites, pleural effusion, encephalopathy and hepatorenal syndrome. We identified the presence or absence of these comorbidities and cirrhosis-related complications for each patient by querying the Taiwan NHI database using the International Classification of Diseases 9 codes (ICD-9). Any cirrhosis-related complication existed before the admission for surgery was defined as pre-operative cirrhosis-related complication. It was identified from the ICD-9 codes for inpatients within 6 months before surgery. Post-operative cirrhosis-related complication was identified from the ICD-9 codes for each discharge from surgery in the cohort.

This study used income-related insurance payment amount as a proxy measure of individual SES, which is an important prognostic factor for survival [17],[18]. The individuals were classified into three groups: (1) low SES, lower than US$528 per month (New Taiwan Dollars (NT) 0, $1 to $15,840), (2) moderate SES, between US$528 to $833 per month (NT $15,841 to $25,000), and (3) high SES, US$833 per month (NT $25,001) or more [19]. We selected NT$15,840 as the low income level cutoff point because this was the government-stipulated minimum wage for full-time employees in Taiwan from 2002 to 2006.

### Statistical Analysis

The SAS statistical package (version 9.2; SAS Institute, Inc., Cary, N.C.) and SPSS (version 15, SPSS Inc., Chicago, IL, USA) were used to analyze the data. A p-value of *P*<0.05 was used to determine statistical significance. The cumulative 30-day, 3-month, and 1-year survival rates and the survival curves were constructed and compared using a log-rank test. Survival was measured from the time after hepatic resection by using overall death as censoring variables. The Cox proportional hazard regression model was used to assess the hospital and the surgeon volume and the combined effects of surgeon and hospital volume on short-term survival after adjusting for patient demographic variables.

## Results

Of all hepatic resections between 2002 and 2006 in Taiwan, 61.2% was for HCC, 25.2% for benign disease, 8.8% for metastatic malignancy and 4.8% for cholangiocarcinoma. Patients’ characteristics are summarized in Table 1. Among these patients, 29.1% of them were operated by high-volume surgeons in high-volume hospitals, 23.1% by low-volume surgeons in high-volume hospitals, 7.7% by high-volume surgeons in low-volume hospitals and 40.1% by low-volume surgeons in low-volume hospitals.

**Table 1:** Baseline characteristics according to hospital volume and surgeon volume (n = 13159).

Variable	High-volume hospital				Low-volume hospital				*P* value
	High-volumesurgeon (n = 3830)		Low-volume surgeon (n = 3035)		High-volume surgeon (n = 1010)		Low-volume surgeon (n = 5284)		
	n (%)		n (%)		n (%)		n (%)		
Age, years (mean ±SD)	55.06±13.95		56.56±15.08		57.17±12.96		58.00±14.50		<0.001
Gender									0.002
Male	2561	(66.9)	1926	(63.5)	637	(63.1)	3347	(63.3)	
Female	1269	(33.1)	1109	(36.5)	373	(36.9)	1937	(36.7)	
Indication for surgery									<0.001
Hepatocellular carcinoma	2667	(69.6)	1718	(56.6)	621	(61.5)	3046	(57.6)	
Cholangiocarcinoma	166	(4.3)	143	(4.7)	51	(5.0)	276	(5.2)	
Metastatic malignancy	159	(4.2)	441	(14.5)	74	(7.3)	486	(9.2)	
Benign disease	838	(21.9)	733	(24.2)	264	(26.1)	1476	(27.9)	
Surgical procedure									0.007
Lobectomy or more	983	(25.7)	732	(24.1)	232	(23.0)	1423	(26.9)	
< Lobectomy	2847	(74.3)	2303	(75.9)	778	(77.0)	3861	(73.1)	
With hepatitis/cirrhosis	1629	(42.5)	1113	(36.7)	423	(41.9)	1992	(37.7)	<0.001
Pre-operative cirrhosis-related complication	352	(9.2)	281	(9.3)	90	(8.9)	679	(12.9)	<0.001
Post-operative cirrhosis-related complication	166	(4.3)	140	(4.6)	47	(4.7)	331	(6.3)	<0.001
Comorbidity									
Hypertension	440	(11.5)	433	(14.3)	135	(13.4)	725	(13.7)	0.003
Ischemic heart disease	38	(1.0)	52	(1.7)	12	(1.2)	95	(1.8)	0.009
Arrhythmia	33	(0.9)	34	(1.1)	5	(0.5)	68	(1.3)	0.066
Heart failure	5	(0.1)	18	(0.6)	3	(0.3)	24	(0.5)	0.012
Cerebrovascular disease	14	(0.4)	23	(0.8)	6	(0.6)	58	(1.1)	0.001
COPD and associated condition	30	(0.8)	31	(1.0)	8	(0.8)	69	(1.3)	0.085
Renal disease	43	(1.1)	83	(2.7)	18	(1.8)	147	(2.8)	<0.001
Diabetes	364	(9.5)	384	(12.7)	125	(12.4)	733	(13.9)	<0.001
Socioeconomic status									<0.001
High	1051	(27.4)	651	(21.4)	220	(21.8)	911	(17.2)	
Moderate	1439	(37.6)	1163	(38.3)	433	(42.9)	2308	(43.7)	
Low	1340	(35.0)	1221	(40.2)	357	(35.3)	2065	(39.1)	
Geographic region									<0.001
Northern	1872	(48.9)	1393	(45.9)	413	(40.9)	2311	(43.7)	
Central	730	(19.1)	591	(19.7)	185	(18.3)	922	(17.4)	
Southern/Eastern	1228	(32.1)	1045	(34.4)	412	(40.8)	2051	(38.8)	
Urbanization level of residence									<0.001
Urban	1212	(31.6)	1013	(33.4)	298	(29.5)	1188	(22.5)	
Suburban	1586	(41.4)	1253	(41.3)	421	(41.7)	2254	(42.7)	
Rural	1032	(26.9)	769	(25.3)	291	(28.8)	1842	(34.9)	

Abbreviation: SD, standard deviation; COPD, chronic obstructive pulmonary disease.

**Figure 1:**
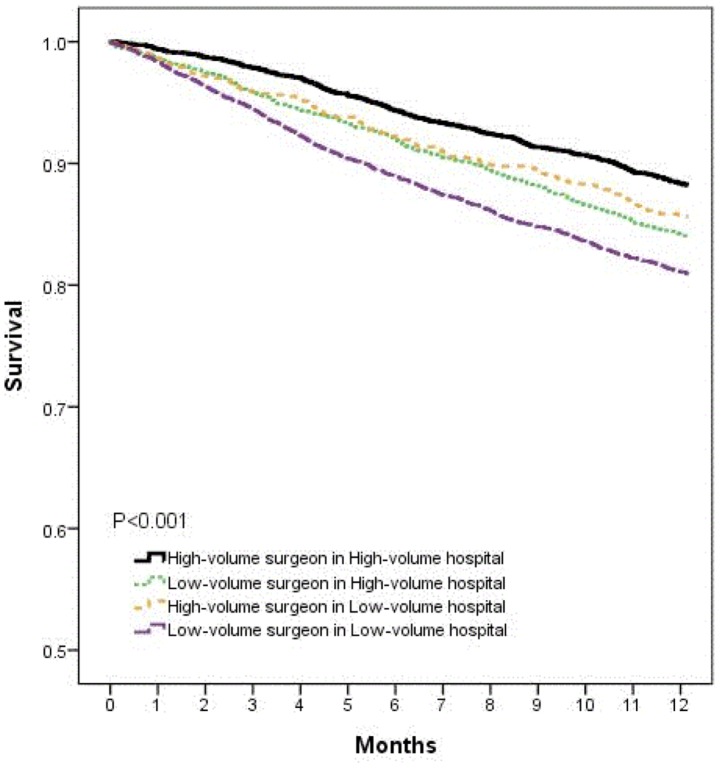
Short-term survival after hepatic resection by combined effect of surgeon and hospital volume.

Figure 1 shows the Kaplan-Meier survival probabilities after hepatic resection in each volume group. The high-volume surgeon in high-volume hospital had the highest survival and the low-volume surgeon in low-volume hospital had the lowest.

Table 2 shows the adjusted hazard ratios based on the Cox proportional hazards regression models after adjusting for patients’ age, gender, indication for surgery, surgical procedure, comorbidity, hepatitis/cirrhosis status, presence of pre-operative and post-operative cirrhosis-related complication, socioeconomic status, geographic region, and urbanization level of residence. In model 1, examining the relationships between hospital volume and short-term survival; after adjusting for other factors, low-volume hospitals had the hazard ratio of 1.50 (95% CI, 1.09–2.07) in 30-day mortality, 1.56 (95% CI, 1.30–1.86) in 3-month mortality and 1.33 (95% CI, 1.21–1.46) in 1-year mortality compared to high-volume hospitals. In model 2, after adjusting for other factors, the relationships between surgeon volume and short-term survival showed that low-volume surgeons had the hazard ratio of 1.64 (95% CI, 1.12–2.41) in 30-day mortality, 1.62 (95% CI, 1.31–2.00) in 3-month mortality and 1.41 (95% CI, 1.27–1.56) in 1-year mortality compared to high volume surgeons. In model 3, examining the combined effects of hospital and surgeon volume, after adjusting for other factors, low-volume surgeons in high- and in low-volume hospitals were associated with higher 30-day mortality; the hazard ratios were 1.81 (95% CI, 1.06–3.08) and 2.15 (95% CI, 1.33–3.46) respectively compared to high-volume surgeons in high-volume hospitals. In 3-month mortality, patients not operated by high-volume surgeons in high-volume hospitals had the hazard ratios ranging from 1.66 to 2.08 (p<0.001). In 1-year mortality, patients not operated by high-volume surgeons in high-volume hospitals had the hazard ratios ranging from 1.28 to 1.58 (p<0.01).

**Table 2:** The adjusted hazard ratios of provider categories in different regression models.

Variable	30-day mortality risk			3- month mortality risk			1-year mortality risk		
	HR	95% CI	*P* value	HR	95% CI	*P* value	HR	95% CI	*P* value
Model 1									
High-volume hospital	1			1			1		
Low-volume hospital	1.50	1.09–2.07	0.013	1.56	1.30–1.86	<0.001	1.33	1.21–1.46	<0.001
Model 2									
High-volume surgeon	1			1			1		
Low-volume surgeon	1.64	1.12–2.41	0.010	1.62	1.31–2.00	<0.001	1.41	1.27–1.56	<0.001
Model 3									
High-volume surgeon inHigh-volume hospital	1			1			1		
Low-volume surgeon inHigh-volume hospital	1.81	1.06–3.08	0.028	1.66	1.24–2.22	0.001	1.33	1.16–1.53	<0.001
High-volume surgeon inLow-volume hospital	1.98	0.99–3.96	0.051	1.90	1.30–2.79	0.001	1.28	1.05–1.56	0.013
Low-volume surgeon inLow-volume hospital	2.15	1.33–3.46	0.002	2.08	1.61–2.69	<0.001	1.58	1.40–1.78	<0.001

Abbreviation: HR, hazard ratio; 95% CI, 95% confidence interval.

Adjusted for patients’ age, gender, indication for surgery, surgical procedure, comorbidity, hepatitis/cirrhosis, pre-and post-operative cirrhosis-related complication, socioeconomic status, geographic region and urbanization level of residence.

Table 3 demonstrates other factors associated with short-term survival at three different time points after hepatic resection. Those had a negative influence on survival included increased age, undergoing major surgical procedure (lobectomy or more), presence of cirrhosis-related complication, ischemic heart disease, renal disease and with a low SES. Cholangiocarcinoma and metastatic malignancy had a poor outcome than HCC.

**Table 3:** The adjusted hazard ratios of patient demographic variables.

Variable	30-day mortality risk			3- month mortality risk			1-year mortality risk		
	HR	95% CI	*P* value	HR	95% CI	*P* value	HR	95% CI	*P* value
Age, year	1.00	0.99–1.01	0.541	1.01	1.00–1.02	0.001	1.008	1.004–1.01	<0.001
Gender									
Male	1			1			1		
Female	0.94	0.67–1.32	0.728	1.04	0.86–1.26	0.640	1.05	0.95–1.16	0.306
Indication for surgery									
Hepatocellular carcinoma	1			1			1		
Cholangiocarcinoma	0.89	0.45–1.76	0.743	1.52	1.13–2.06	0.005	2.06	1.78–2.40	<0.001
Metastatic malignancy	1.20	0.65–2.19	0.556	1.34	0.98–1.82	0.060	1.34	1.14–1.56	<0.001
Benign disease	0.88	0.56–1.38	0.599	0.49	0.36–0.66	<0.001	0.27	0.22–0.32	<0.001
Surgical treatment									
Lobectomy or more	1			1			1		
< Lobectomy	0.66	0.47–0.93	0.019	0.71	0.59–0.86	0.001	0.64	0.58–0.71	<0.001
With hepatitis/cirrhosis	1.29	0.90–1.84	0.160	1.01	0.83–1.23	0.876	0.92	0.83–1.02	0.156
Pre-operative cirrhosis-related complication	0.44	0.16–1.20	0.110	3.11	2.43–3.98	<0.001	5.93	5.29–6.64	<0.001
Post-operative cirrhosis-related complication	7.22	2.53–20.61	<0.001	1.32	0.98–1.79	0.065	0.44	0.37–5.33	<0.001
Comorbidity									
Hypertension	0.85	0.51–1.41	0.536	0.63	0.47–0.85	0.003	0.71	0.61–0.83	<0.001
Ischemic heart disease	3.87	1.99–7.52	<0.001	2.73	1.76–4.22	<0.001	1.67	1.24–2.26	0.001
Arrhythmia	1.87	0.68–5.16	0.224	0.92	0.43–1.96	0.842	0.76	0.49–1.18	0.226
Heart failure	1.59	0.48–5.21	0.440	1.95	0.98–3.88	0.055	1.83	1.05–3.19	0.031
Cerebrovascular disease	1.57	0.38–6.41	0.527	2.77	1.51–5.07	0.001	1.43	0.91–2.27	0.118
COPD and associated condition	2.67	0.97–7.32	0.056	1.02	0.48–2.16	0.956	1.24	0.85–1.81	0.258
Renal disease	8.96	5.95–13.47	<0.001	5.11	3.89–6.70	<0.001	2.62	2.14–3.21	<0.001
Diabetes	0.91	0.56–1.45	0.694	1.05	0.82–1.35	0.684	0.92	0.80–1.06	0.306
Socioeconomic status									
Low	1			1			1		
Moderate	0.78	0.55–1.11	0.183	1.05	0.87–1.29	0.569	1.14	1.02–1.27	0.014
High	0.40	0.22–0.73	0.003	0.42	0.30–0.59	<0.001	0.77	0.67–0.89	0.001
Geographic region									
Northern	1			1			1		
Central	1.65	1.08–2.51	0.019	1.18	0.92–1.50	0.174	1.05	0.93–1.20	0.389
Southern/Eastern	0.91	0.61–1.35	0.647	0.85	0.68–1.05	0.137	0.89	0.80–1.01	0.060
Urbanization level of residence									
Urban	1			1			1		
Suburban	1.11	0.72–1.71	0.633	0.89	0.71–1.12	0.337	0.96	0.85–1.09	0.586
Rural	1.49	0.93–2.37	0.091	0.99	0.77–1.28	0.987	1.04	0.91–1.19	0.500

Abbreviation: HR, hazard ratio; 95% CI, 95% confidence interval; COPD, chronic obstructive pulmonary disease.

## Discussion

The current study from a national database identifying 13,159 hepatic resections between 2002 and 2006 in Taiwan revealed that short-term survival after hepatic resection was largely influenced by the combined effects of hospital and surgeon volume. Surgeon volume had a significant impact on 30-day mortality, high-volume surgeons in high- and in low-volume hospitals demonstrated better outcomes than low-volume surgeons. When looking at 3-month and 1-year mortality, high-volume surgeons in high-volume hospitals demonstrated a significantly better outcome than other three.

The strength of this study are based on the fact that it was a nationwide population-based cross-sectional study, include almost all patients undergoing hepatic resection in Taiwan. At the end of 2006, the NHI covered 99.0% of Taiwan’s population [14], with nearly complete follow-up information of mortality among the whole study population, as well as the fact that the dataset was routinely monitored for diagnostic accuracy by the National Health Insurance Bureau of Taiwan. The current study not only took most of the known prognostic factors into account but also analyzed short-term survival at three time points after hepatic resection. Furthermore, hepatic resection had not been centralized in Taiwan to a significant degree during the study period, which allowed for investigation of the effect of volume.

Patient-related difference is important in the volume-outcome relationship study. Some studies revealed the minority, older, and low SES patients are more likely to be treated at low-volume hospitals [20],[21]. And there is a negative association between SES and cancer survival rate [22],[23],[24]. A study from the United States also revealed that patients who underwent cancer operations for lung, esophagus, and pancreas tumors with more comorbidities were more likely to receive their cancer surgery at low-volume hospitals [25]. Although these trends were also seen in this current study, several of these variables were associated with increased short-term mortality and were entered into the multivariate analysis. After adjusting for case-mix in this fashion, there were no changes in the trends for mortality that low-volume providers had inferior outcomes.

A great body of literature has addressed the relation between hospital volume for hepatic resection and mortality. Most previous studies reported a substantial inverse relation between hospital volume and mortality, but the roll of the surgeon volume was not fully established yet. A study from the United States reported that the surgeon volume for hepatic resection was not associated with in-hospital mortality [9], but the present study found contradictory results. In that study, it did not adjust for the known prognostic factors, eg, indication for surgery, hepatitis/cirrhosis, cirrhosis-related complications, socioeconomic status, and lacked an analysis for the combined effect of hospital and surgeon volume.

In agree with previous studies of volume-outcome relationship in some other procedures, surgeon volume was inversely associated with short-term mortality [3],[4],[6]. However, this association did not remain in 3-month and 1-year mortality after hepatic resection for high-volume surgeons in low-volume hospitals after taking hospital volume into account. This finding indicates that hospital-volume factors, eg, more specialties for perioperative care of liver diseases, skilled nursing staff and developed intensive care units, account for the effect of surgeon volume on short-term mortality, might influence the risk of mortality after hepatic resection.

In line with previous studies, high-volume hospital or high-volume surgeon was a predictor of survival [7],[8],[9]. In the present study, it provides an additional insight on the volume-outcome relationship based on the combination of surgeon and hospital volume that high-volume surgeons in high-volume hospitals was a predictor of short-term survival after hepatic resection; high-volume surgeons in low-volume hospitals as well as low-volume surgeons in high-volume hospitals were not. Nevertheless, the positive association remained in high-volume surgeons when comparing with low-volume surgeons. These results indicated the effect of surgeon volume on patients’ short-term survival after hepatic resection. Surgeons should maintain a higher volume of hepatic resections and increase experience for a better outcome.

It also has been suggested that the referral of patients to high-volume hospitals will provide a better application of recommended processes of care [2],[26]. In the present study, patients who underwent hepatic resection by low-volume surgeons in high-volume hospitals had an increased risk of mortality compared to those who underwent hepatic resection by high-volume surgeons in high-volume hospitals. Our finding suggests that in addition to referring patients to a high-volume hospital, referring patients to a high-volume surgeon in high-volume hospital may be another important consideration.

There are some limitations to this study. First, diagnosis and any comorbidity were completely dependent on ICD codes, any coding errors in patients’ underlying diseases could lead to disparities in comorbidity and cirrhosis-related complication. Nonetheless, the National Health Insurance Bureau of Taiwan randomly reviews the charts and interviews patients in order to verify diagnostic accuracy [27]. Second, instead of surgical mortality, the all-cause mortality was used. But the short-term surgical and all-cause mortality differ only to a small degree and it is unlikely that the differences would be systemically different for high- and low-volume providers. Third, the cutoff used for assessing surgeon volume can be considered a little low; however, they are comparable to those of previous large studies that addressed hospital and surgeon volume from the United States [7],[8],[28].

In summary, our findings provide supports for the combined effects of hospital and surgeon volume with regard to short-term survival after hepatic resection. This population-based study revealed that short-term survival after hepatic resection was largely influenced by the combined effects of hospital and surgeon volume. There was a clear association between high-volume surgeons in high-volume hospitals and better short-term survival. Centralization of hepatic resection to few surgeons and hospitals might improve patients’ prognosis.

## Supporting Information

Appendix S1 **The process of defining the hospital and the surgeon volume.** Table S1, defining the category of hospital volume. Table S2, defining the category of surgeon volume.
(DOC)
