# Supplementary material for: The Combined Effects of Hospital and Surgeon Volume on Short-Term Survival after Hepatic Resection in a Population-Based Study
Source: PLoS One. 2014 Jan 22;9(1):e86444. doi: 10.1371/journal.pone.0086444 (PMC3899267; doi:10.1371/journal.pone.0086444)
Supplement: Appendix S1 — The process of defining the hospital and the surgeon volume. Table S1, defining the category of hospital volume. Table S2, defining the category of surgeon volume. (DOC) [file pone.0086444.s001.doc]

**Supporting Information**

**Appendix S1**

1. The caseload of each hospital and surgeon was calculated.

2. Sorting the hospital’s and surgeon’s order by caseload number.

3. Defining the category of caseload was as the following steps.

Table S1. The process of defining the category of hospital volume.

| Hospital ID | Caseload | Cumulative case |
| --- | --- | --- |
| 1 | 1 | 1 |
| 2 | 1 | 2  Roughly  1/2 cases of  hepatic resections |
| 3 | 1 | 3  Low volume |
| 4 | 2 | 5 |
| . |  |  |
| . |  |  |
| 363 | 238 | 6294 |
| 364 | 245 |  |
| 365 | 269 | Roughly  1/2 cases of  hepatic resections |
|  |  | High volume |
|  |  |  |
| 375  376 | 1171  1269 | 13159 |

| Table S2. The process of defining the category of surgeon volume. | | |
| --- | --- | --- |
| Surgeon ID | Caseload | Cumulative case |
| 1 | 1 | 1 |
| 2 | 1 | 2 |
| 3 | 2 | 4 |
| 4 | 2 | 6 |
| . |  |  |
| . |  |  |
| 3322 | 24 | 8319 |
| 3323 | 25 |  |
|  |  |  |
|  |  |  |
|  |  |  |
| 3380  3381 | 377  474 | 13159 |

Roughly

2/3 cases of

hepatic resections

Low volume

Roughly

1/3 cases of

hepatic resections

High volume
